# Identifying key regulators of the intestinal stem cell niche

**DOI:** 10.1042/BST20210223

**Published:** 2021-10-19

**Authors:** Carrie A. Duckworth

**Affiliations:** Henry Wellcome Labs of Cellular and Molecular Gastroenterology, Department of Molecular Physiology and Cell Signalling, Institute of Systems, Molecular and Integrative Biology, University of Liverpool, Crown St, Liverpool L69 3GE, U.K.

**Keywords:** epithelial dynamics, gastrointestinal physiology, intestinal epithelial cells, intestinal homeostasis, intestinal stem cell niche, intestinal stem cells

## Abstract

The intestinal tract is lined by a single layer of epithelium that is one of the fastest regenerating tissues in the body and which therefore requires a very active and exquisitely controlled stem cell population. Rapid renewal of the epithelium is necessary to provide a continuous physical barrier from the intestinal luminal microenvironment that contains abundant microorganisms, whilst also ensuring an efficient surface for the absorption of dietary components. Specialised epithelial cell populations are important for the maintenance of intestinal homeostasis and are derived from adult intestinal stem cells (ISCs). Actively cycling ISCs divide by a neutral drift mechanism yielding either ISCs or transit-amplifying epithelial cells, the latter of which differentiate to become either absorptive lineages or to produce secretory factors that contribute further to intestinal barrier maintenance or signal to other cellular compartments. The mechanisms controlling ISC abundance, longevity and activity are regulated by several different cell populations and signalling pathways in the intestinal lamina propria which together form the ISC niche. However, the complexity of the ISC niche and communication mechanisms between its different components are only now starting to be unravelled with the assistance of intestinal organoid/enteroid/colonoid and single-cell imaging and sequencing technologies. This review explores the interaction between well-established and emerging ISC niche components, their impact on the intestinal epithelium in health and in the context of intestinal injury and highlights future directions and implications for this rapidly developing field.

## Introduction

**Figure 1.**
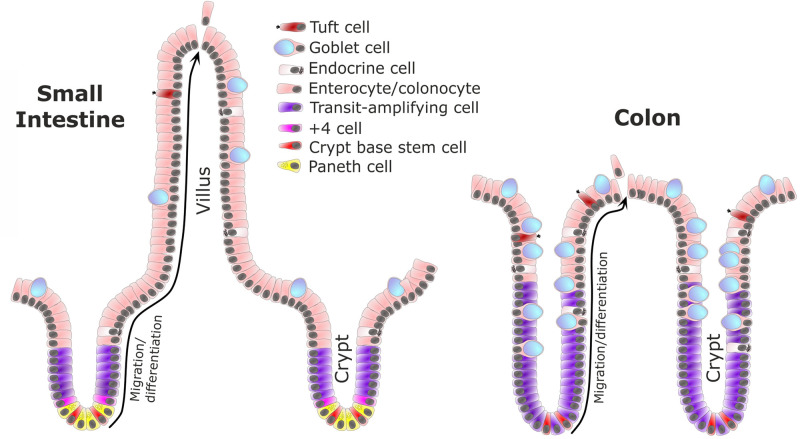
The small intestinal epithelium forms crypt and villus structures overlying the lamina propria (left) whereas the colonic epithelium only has crypts (right). Active cycling intestinal stem cells (ISCs) are located within the crypt base (red). Paneth cells (yellow) are only found in the small intestine. Under homeostatic conditions, ISCs divide via a neutral drift process to generate either two ISCs or two cells that enter the transit-amplifying population (purple) which maintain a limited capacity to divide prior to maturation into secretory or absorptive lineages [5,19]. Cells (other than ISCs and Paneth cells which are longer lived and remain in the crypt base) undergo a process of migration and differentiation along the crypt-villus axis (black arrow). Once epithelial cells reach the villus tip in the small intestine or inter-crypt table in the colon, they undergo apoptosis and are shed into the lumen. Neighbouring cells reform tight junctions beneath the shedding cell to ensure the intestinal epithelial barrier is maintained throughout the extrusion process [5,92,108]. The process from ISC division at the crypt base to apoptosis and cell shedding at the villus tip takes 3–5 days depending on species and location along the cephalocaudal axis [109].

The luminal surface of the gastrointestinal tract is covered by a single layer of epithelial cells that forms a continuous physical barrier to intestinal contents that include microbiota and dietary factors. Disruption of this physical barrier can lead to infection, inflammation and in severe cases, sepsis [1,2]. The epithelium forms crypts and villi in the small intestine and crypts in the colon (Figure 1). Crypt-villus axes are important structures for massively enhancing the surface area of the gut for efficient absorption, but which need to be tightly regulated to ensure that a sufficient and appropriate epithelial cell population is maintained [3–5]. The mechanisms controlling homeostasis of the intestinal mucosa and particularly epithelial barrier maintenance in health and during injury and restitution have been the focus of research for many decades. However, recent technologies such as intestinal organoid, reconstituted organoid, enteroid and colonoid cultures, single sell sequencing and higher resolution imaging are currently enabling further understanding of this complex heterogeneous autoregulatory system in much finer detail.

Actively cycling intestinal stem cells (ISCs) are located within the crypt base of the small intestine and colon [6]. A population of quiescent, reserve stem cells that have capacity to re-populate intestinal epithelia upon intestinal injury are also found around four cell positions from the crypt base [7,8]. However, recent studies have assessed the plasticity of the epithelium and have shown that under certain conditions, cells previously thought to be terminally differentiated are able to de-differentiate to replenish lost ISCs [9]. Mechanisms that regulate proliferation, apoptosis, migration and differentiation of intestinal epithelial cells have been well characterised, however, questions still remain as to how all of these processes are co-ordinated to achieve equilibrium during the perpetual state of epithelial cell renewal observed in the intestine. Factors that control the ISC niche are produced by ISCs themselves, neighbouring Paneth cells and specialised sub-epithelial mesenchymal cells including pericryptal myofibroblasts, telocytes, newly identified trophocytes and immune cells [10–12]. Additional non-cellular components of the ISC niche include the basement membrane matrix. Feedback mechanisms are crucial along the entire crypt-villus axis to regulate ISC niche activities. Biochemical gradients are established that sustain the ISC niche but continue to drive cells through various states of specialisation once they leave the stem cell zone [11,13–15]. Recent studies have greatly improved our understanding of the contribution of gradients, such as those produced by Wnt and Bmp ligands, to different cellular activities, although further elucidation is now needed to fully understand the complexities of interaction between different cellular compartments and their local environments.

## Key intestinal stem cell niche components

### Stem cells and epithelial neighbours

ISCs are maintained as ISCs by the physical niche they reside within that comprises of direct ligand-mediated contact with other cell populations and the extracellular matrix, chemokine, cytokine and growth factor signalling. Once the niche becomes unfavourable for stem cell activity, ISC phenotype can change and stemness is lost. Several markers of active ISC populations under homeostatic conditions have been defined (including Lgr5, Ascl2, Olfm4). Six-16 Lgr5^+^ ISCs populate a single small intestinal crypt and divide daily following a neutral drift process [16–19]. Several additional markers label both active cycling ISCs and quiescent ISCs including Bmi1, Lrig1, Hopx, mTert, Krt19, Clu, Mex3a, Atoh1 and when expressed can eventually give rise to Lgr5^+^ ISCs after injury-induced depletion of the Lgr5^+^ ISC pool, such as following γ-irradiation, colitis, or during experimental targeted ablation [20,21]. The +4 quiescent ISCs, so called as the name represents their cell position from the crypt base, are undoubtably a population capable of replenishing the active ISC supply if lost. However, recent studies have shown that Paneth cells [22,23], late stage enteroendocrine cells and secretory and absorptive cell progenitors also have the capacity to de-differentiate into an ISC role [24–29] and their chromatin and RNA profiles change to those similar to Lgr5^+^ ISCs after intestinal injury [30] suggesting that the intestinal epithelium has a high degree of plasticity based on local environmental stimuli. De-differentiation was subsequently shown to require the Ascl2 transcription factor that is expressed by epithelial cells prior to their movement into the stem cell niche and expression of Lgr5 [9].

Epithelial-mesenchymal cell crosstalk is thought to contribute to reprogramming epithelial cell identity with a foetal-like phenotype during intestinal regeneration [31]. The capacity of epithelial cells to undergo epithelial-mesenchymal transition (EMT) particularly as a result of the activation of foetal-like programmes also demonstrate cellular and tissue plasticity [32]. Crypts overlying granulomas caused by mucosal invasion of *Heligmosomoides polygyrus* lost Lgr5, Olmf4 and other stem cell-associated expressions, but crypts became hyper-proliferative and less differentiated as a result of an IFNγ-dependent transcriptional programme that was enriched in genes normally expressed in the foetal intestinal epithelium such as Sca-1 (Ly6a) [33]. Sca-1 has also been shown to be expressed on epithelia during active colitis with crypt disruption where Lgr5 expression was absent and the Hippo pathway effectors YAP/TAZ were active [33–35]. Sca-1^+^ intestinal organoids grew as spheroids without the need for the addition of Wnt or R-spondin to culture media whereas Sca-1^−^ intestinal organoids had a normal budding phenotype and normal Wnt activator requirements, suggesting that Sca-1 could mark a population of reserve stem cells following some forms of intestinal injury [33]. Expression of foetal organoid enriched genes such as Anxa1 and Tacstd2/Trop are up-regulated within the regenerating intestinal epithelium [35] and differences in disease characteristics between right versus left sided colon cancer pathogenesis may be attributed to Lgr5^+^ versus Lgr5^−^ Sca-1^+^ ISC populations [36]. Epithelial cells in the ISC niche may therefore display region specific differences along the cephalocaudal axis leading to spatial modulation of the ISC niche. Care should be taken in ISC niche studies to document the precise anatomical location and inflammation/regeneration status of the intestinal mucosa.

Paneth cells are derived from quiescent secretory progenitor cells at the base of the transit-amplifying region, are long lived and are the only differentiated cell population to migrate towards the crypt base where they directly interact with ISCs [24]. Intestinal organoid studies have allowed the assessment of the contribution of Paneth cells to ISC maintenance [37]. Paneth cells produce and secret Wnt3, EGF and have membrane bound Dll1 and 4 that can activate MAPK, EGFR and Ras signalling along with Wnt and Notch pathways which enables them to enhance ISC growth [37]. However, recent studies have demonstrated that Paneth cells are not a requirement to sustain the ISC niche *in vivo* and other ISC niche components can substitute for their activity [38,39]. cKit^+^/Reg4^+^ cells have been identified in colonic crypt bases and are thought to be colonic Paneth cell equivalents with the exception that the cKit^+^/Reg4^+^ population do not produce Wnt ligands [40,41]. Wnt and Notch signalling pathway components that supplied to the ISC niche by Paneth cells are discussed below.

### Stem cells and mesenchymal neighbours

Many different cell populations form or infiltrate the intestinal lamina propria and each cell type generates membrane tethered ligands and a secretome that could potentially impact on the ISC niche. Telocytes have recently been identified as key controllers of the ISC niche [42–44] but share several features with sub-epithelial myofibroblasts (SEMFs). Due to recent advances in imaging, single-cell sequencing, and the identification of various sub-populations and locations of these cell types, the nomenclature that categorises these populations as discrete is still being defined [45,46]. Telocytes are thought to be distinct from SEMFs, smooth muscle and interstitial cells of Cajal (ICC) in that they express CD34 [47], PDGFRα [48], the Foxl1 transcription factor [42] and in the colon Gli1 [44], but don't express Acta2 (α-SMA), Myh11 [42] or c-Kit [49]. SEMFs express Myh11 and Acta2 but are located similarly to telocytes adjacent to the intestinal epithelium and a sub population also express the telocyte markers Foxl1, Gli1 [46] and PDGFRα [50]. SEMFs have been shown to regulate intestinal and gastric organoid growth and differentiation [51,52] *in vitro* and secrete factors that stimulate epithelial cell proliferation *in vivo* [53]. Signalling from SEMFs controls various aspects of the ISC niche and differentiation programme, however, there is functional redundancy with other mesenchymal and epithelial cell populations in their role as ISC niche regulators. For instance, blocking Wnt secretion from SEMFs does not appear to affect ISC proliferation or differentiation [54]. Due to heterogeneity within telocyte and SEMF populations, it is important to define these cells based on expression profiles until clear functional distinctions are made. There are also species differences in expression patterns and different anatomical regions of the gut that require further characterisation. Holloway et al. [55] have defined a sub-epithelial cell that lines the entire crypt-villus axis and expresses DLL1, F3 and PDGFRα. This population can be further subdivided into those found in the villus that express NPY and those found in the crypt that are NPY− [55]. Populations of sub-epithelial cells in the human colon express WNT5A, WNT5B, BMP2 and BMP4 [56].

**Figure 2.**
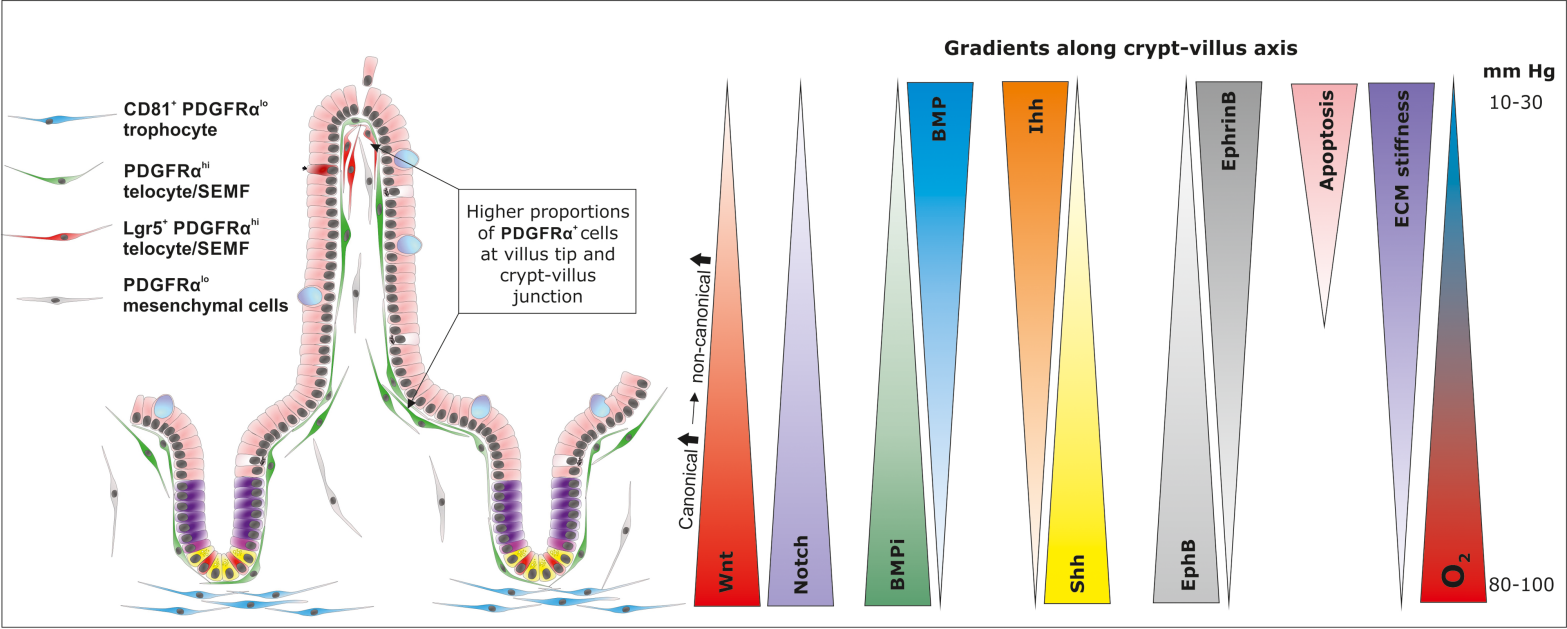
Mesenchymal cells form a continuous network along the crypt-villus axis. Trophocyte, telocyte and sub-epithelial myofibroblasts (SEMF) are key cell populations that regulate signalling gradients along the crypt-villus axis to promote the ISC niche at the crypt base and differentiation programmes at higher cell positions. Trophocytes express CD81 and predominate towards the crypt base (blue) and telocytes/SEMFs span the crypt base to villus tip with higher abundance observed at the crypt-villus junction and villus tip (red/green). The expression patters of telocytes along the crypt-villus axis is non-uniform and due to the heterogeneity of this cell population identified from single cell sequencing, discrete sub-populations are currently difficult to identify. Telocytes express higher amounts of PDGFRα than trophocytes and a sub-population of Lgr5 expressing telocytes are located within the villus tip (red). Signalling gradients that contribute to ISC niche regulation are indicated.

A critically important population of mesenchymal cells required to maintain ISC proliferation are termed ‘trophocytes’ and have recently been defined as expressing CD81 and lower amounts of PDGFRα (PDGFRα^lo^) than telocytes and they are localised to the lamina propria specifically underlying the intestinal crypt base [11] (Figure 2). Trophocytes were required for epithelial cell growth in intestinal organoid culture (without exogenous R-spondin) as they produce R-spondins and BMP inhibitors including Grem1. Conversely, in the same reconstituted organoid system telocytes were shown to produce large amounts of BMP which inhibited organoid growth [11]. Direct interaction of telocyte processes with intestinal epithelial cells have been observed [57] and with these processes spanning several epithelial cells in length, they form a mesh-like peri-cryptal sheath beneath the epithelium [58]. Plasticity between mesenchymal cell populations is thought to occur during remodelling of the epithelium upon recovery from intestinal injury [59]. We must also be mindful of the potential expansion and differentiation of mesenchymal cell populations within the lamina propria. In an elegant study conducted by Worthley et al., Grem1 was conditionally labelled in mice and lineage tracing of Grem1^+^ cells demonstrated that they were able to expand in number slowly and migrate to form several mesenchymal lineages (Acta2^+^ myofibroblasts and Acta2^−^, Ng2^+^ cells) along the entire crypt-villus sheath which led to the identification of this population as intestinal reticular stem cells (iRSCs) that can give rise to multiple mesenchymal cell types [60]. Ng2 (Cspg4) labels both pericytes and Foxl1 positive and negative telocytes [61].

### Key signalling pathways that maintain the intestinal stem cell niche

#### Wnt signalling

**Figure 3.**
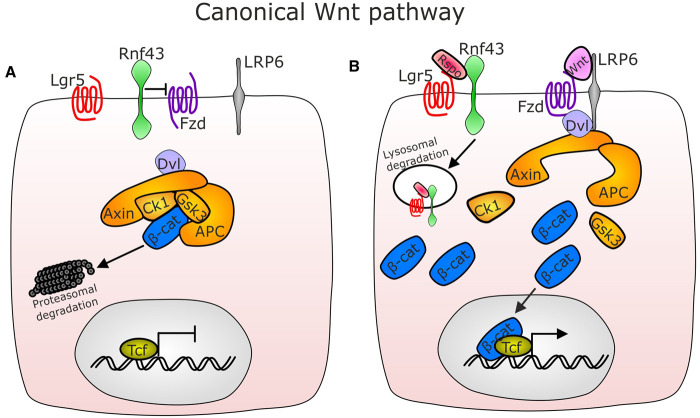
Canonical Wnt signalling is critical for ISC niche maintenance and is potentiated by Lgr5. In the absence of Wnt ligands, β-catenin is held in a destruction complex that includes Dvl, Axin, Ck1, Gsk3 and Apc which results in β-catenin being targeted to the proteasome for degradation (**A**). When present, canonical Wnt ligands (eg Wnt3a) bind to frizzled receptors (Fzd) on proliferation-permissible cells which then recruit LRP6 and Dvl and release β-catenin from the destruction complex allowing β-catenin stabilisation and cytoplasmic accumulation. β-catenin can then translocate to the nucleus and binds to the T-cell factor/lymphoid enhancer factor (Tcf/Lef) transcription factor which results in the activation of a panel of genes critical for cell division [110]. R-spondins (Rspo) are important in potentiating this pathway by binding to Lgr5 and recruiting the Rnf43 ubiquitin ligase which targets the Lgr5–Rspo–Rnf43 complex for lysosomal degradation thus preventing Rnf43 from removing Fzd from the cell membrane and stabilising Fzd to enhance its availability for Wnt ligands [62] (**B**).

Proliferation of Lgr5^+^ ISCs is dependent on Wnt ligands and R-spondin (RSPO) co-factors [62]. Wnt signalling components can be broadly divided into canonical (signalling via β-catenin) and non-canonical (β-catenin-independent) pathways. The canonical Wnt signalling pathway is active within ISCs and proliferative transit-amplifying cells (Figure 3) which respond to Wnt3, Wnt4, Wnt6 and Wnt9b [63]. Canonical Wnt3a is produced by Paneth cells and Wnt2b and R-spondins 1–3 are produced by trophocytes which are in direct contact with ISCs [11]. Wnt2b was able to rescue Wnt3a^−/−^ enteroids demonstrating the importance of this family member in ISC niche maintenance, but also further demonstrating functional redundancy between Wnt family members [39]. Enteroids have also been maintained in co-culture with intestinal mesenchyme without the requirement of additional growth factors [51,64]. R-spondin 3 has a higher potency than R-spondin 1 for ISC niche maintenance and single-cell sequencing suggests that the majority of Wnt and BMP is produced by PDGFRα^+^ cells [11,50]. Telocytes are not the major source of Rspo ligands, however, non-telocytes including trophocytes express R-spondin 3 [43,45,50,61]. Frizzled 7 (Fzd7) has been identified as the key Wnt receptor in Lgr5^+^ ISCs and is critical for maintaining intestinal crypt homeostasis and during epithelial regeneration [65]. The non-canonical Wnt pathway is β-catenin-independent and is not thought to be activated in intestinal epithelial cells. However, recent studies have described its importance in mesenchymal cell regulation and activity which ultimately impact on the ISC niche. Non-canonical Wnt4, Wnt5a and Wnt5b are produced by intestinal telocytes and Wnt9a which can activate canonical and non-canonical Wnt signalling is produced by trophocytes [11]. Wnt/Notch signalling crosstalk are also implicated in secretory cell fate decisions. Wnt inhibitor dickkopf1 (Dkk1) results in a loss of all secretory cell populations along the crypt-villus axis [66].

#### BMP signalling

BMP is a member of the transforming growth factor-β (TGF-β) superfamily and can activate SMAD, p38, JNK and PI3K signalling which when perturbed can result in intestinal cancers [67,68]. BMP signalling determines stemness and cell fate and the establishment of BMP gradients is necessary for homeostatic control of the crypt-villus axis (discussed see section on peri-cryptal telocytes as gradient regulators below). BMP 2 and 4 and BMP inhibitors (BMPi) Chrd and gremlin 1 are expressed by several different cell types along the crypt-villus axis including sub-epithelial trophocytes [11]. BMP acts on epithelial cells via BMPR1A receptors [69]. Epithelial BMP signalling was shown to dampen Lgr5+ ISC renewal which prevented crypt hyperproliferation and intestinal polyp formation in mice [70].

#### Notch signalling

Notch signalling regulates ISC renewal and epithelial cell fate in co-ordination with Wnt signalling. When Notch signalling is repressed, epithelial cells undergo a programme of differentiation into secretory lineages at the expense of Lgr5^+^ ISC, transit-amplifying cells and absorptive lineages [71]. Paneth cells in the small intestine and deep crypt epithelial cells in the colon express the Notch ligands Dll1 and Dll4 but these can also be provided by enteroendocrine and tuft cells in the event of Paneth cell depletion [38]. Dll1 and Dll4 ligands are required for the maintenance of Lgr5^+^ stem cells and the proliferative compartment and their actions are mediated via direct cell-cell contact [71]. Transit-amplifying cells differentiate to absorptive cells following active Notch signalling in a low Wnt environment. When neighbouring secretory cells express Notch ligands, lateral inhibition occurs which helps regulate absorptive and secretory cell proportions [72].

#### Hippo signalling

**Figure 4.**
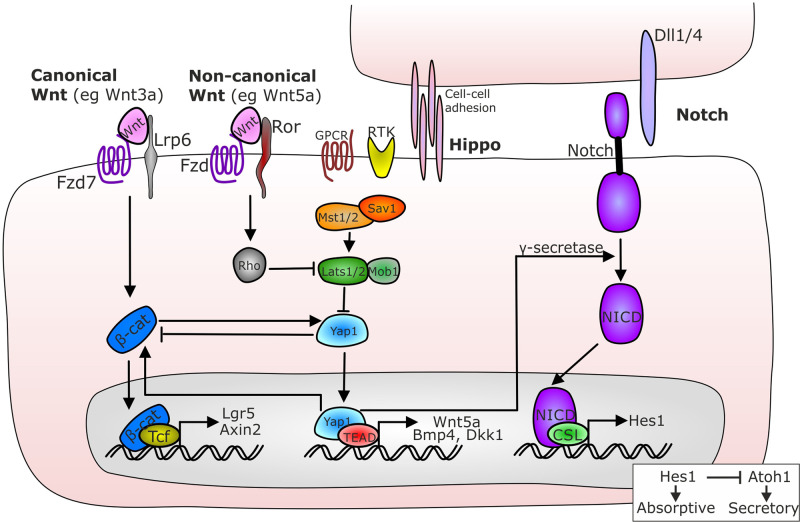
Crosstalk between canonical and non-canonical Wnt, Hippo and Notch signalling pathways. Non-canonical Wnt (eg Wnt 5a) bridges frizzled receptors with Ror to activate Rho kinase that prevents Lats1/2 inhibition of Yap1 resulting in the suppression of β-catenin nuclear translocation and canonical Wnt pathway inhibition. Hippo signalling is modulated by G protein coupled receptors (GPCR), receptor tyrosine kinases (RTK) and by cell-cell adhesion. When Hippo signalling is active, cytosolic Yap1 is also high and inhibits β-catenin translocation. When Hippo signalling is supressed, Yap1 can translocate to the nucleus and binds to TEAD transcription factors which results in the production of non-canonical Wnt 5a, Bmp4 and Dkk1 and also stimulates the nuclear translocation of β-catenin, thus activating canonical Wnt signalling. Nuclear Yap1 also stimulates the activation of Notch signalling by promoting the cleavage of the Notch intracellular domain (NICD) which can bind the CSL transcription factor in the nucleus resulting in production of Hes1. Hes1 represses Atoh1 and modulates whether cells become secretory or absorptive populations.

Whilst Hippo effectors Yap and Taz have been demonstrated as important pathway components in regulating Lgr5-independent crypt regeneration following intestinal injury [73], their role in regulating normal intestinal homeostasis has been debated. A recent study by Li et al. [74] has demonstrated a critical role for the core Hippo kinases Lats1/2 in sustaining Wnt pathway signalling in the ISC niche (Figure 4). Deletion of Lats1/2 resulted in the loss of Lgr5^+^ stem cells and a concomitant rapid Wnt-independent expansion of the transit amplifying population [74].

#### Growth factor signalling

Several ErbB family members (of which epidermal growth factor receptor (EGFR)/ErbB1 is one) are found in multiple cell populations along the crypt-villus axis and are major drivers of proliferation (particularly EGFR) and cell survival in the intestinal crypt. Epidermal growth factor (EGF) is produced by several different cell types including Paneth cells in the ISC niche. Single-cell sequencing has recently shown that EGF is also produced by epithelial cells distant from the ISC niche but not by mesenchymal cells [55]. EGF is routinely added to intestinal organoid cultures to enhance their growth [37], however, recent studies have indicated that neuregulin 1 (NRG1) activates ErbB and can replace EGF in enteroid culture resulting in greater cellular diversity [55,75]. Leucine-rich repeats and immunoglobulin-like domain protein 1 (Lrig1) acts to regulate ISC niche size and ISC number by inhibiting ErbB signalling [76]. Ras and mTOR signalling are important downstream effectors of EGFR activation and components of their signalling pathways are often mutated in human colorectal cancers [68,77]. Activation of mutant KRas-G12D has been shown to result in intestinal hyperplasia, goblet cell differentiation and Paneth cell depletion [78] indicating the importance of this small GTPase in maintaining crypt homeostasis. Activation of mTORC1 also results in hyperproliferation of intestinal epithelial cells and the redistribution of Paneth cells away from the crypt base [79]. Whilst Ras and mTOR do not directly regulate ISC number, they are thought to suppress Wnt signalling in intestinal crypts which indirectly modulates ISC number, proliferation and differentiation [80].

#### Hedgehog signalling

Hedgehog (Hh) signalling is mediated by sonic and Indian (Ihh) hedgehog ligands which are differentially expressed along the crypt-villus axis to generate a Hh gradient (Figure 2). Ihh is more abundant at the villus tip with Sh located in greater abundance in the crypt base [81]. Sh deletion in mice results in defective Paneth cells, ER stress and aberrant autophagy [82]. Hh signals are produced by epithelial cells and interact with multiple mesenchymal cell populations to produce soluble Wnt, BMP and cytokines that feed back to regulate the crypt-villus axis. Several transgenic mouse studies have elucidated the importance of Hh signalling and Hh/Wnt crosstalk and these are extensively reviewed recently by Walton and Gumucio [83]. Foxl1 is abundant in telocyte populations and is activated by Hh signalling from the epithelium via Gli binding sites, enabling close communication between the Foxl1^+^ pericryptal mesenchymal sheath and the epithelium to regulate parameters such as mesenchymal cell number, BMP secretion, crypt size, crypt fission and villus height [82,84–86].

### Exit from the intestinal stem cell niche

Differentiation and maturation of intestinal epithelial cells is critical for intestinal homeostasis and mechanisms are required to prevent the over population of ISCs and their immature, proliferative daughters. Bone morphogenic protein (BMP) signalling has been shown to be important in controlling the exit from the ISC niche by regulating cellular differentiation [15] and an increasing gradient of BMP signalling components can be observed from the intestinal crypt base to the villus tip which is in opposition to Wnt signalling components (Figure 2). It is necessary to restrict BMP signalling within the ISC niche to maintain stem cell function. The BMP4 antagonist, noggin, is routinely applied to intestinal organoids to maintain their stemness and recently Gremlin 1 (Grem1) expression in pericryptal mesenchymal CD81^+^ PDGFRα^lo^ trophocytes *in vivo* has been shown to be important in antagonising BMP signalling at the crypt base [11,87]. Mouse studies have indicated that ectopic crypts containing ISCs populate villus structures when BMP signalling is insufficient within villus domains [88]. Despite evidence to support that Wnt and BMP gradients are important in managing the crypt-villus axis, It is not yet clear how intestinal enteroids maintain tissue polarity within culture systems with the absence of external non-epithelial gradient cues. Paneth cells are the only differentiated cell population to migrate towards the crypt base *in vivo* and in standard enteroid culture and differences in Eph receptor/ephrin signalling were shown to be important in determining migration direction [13]. Interaction between Eph receptors and ephrins are mediated by cell-cell contact and produce cellular repulsion and actin cytoskeleton remodelling that could be partially responsible for the migratory action. Proliferative epithelial cells express EphB2 which gradually diminishes at higher cell positions along the crypt-villus axis. Paneth cells and crypt base columnar cells (now shown to be ISCs) express EphB3 which was shown to maintain their crypt base position. A gradual increase in ephrin B was also documented from crypt base to villus tip which in combination with Eph receptors establishes an Eph/ephrin gradient that is proposed to position all cells along the crypt-villus axis [13]. Eph/ephrin signalling gradients may therefore contribute to cellular exit from the ISC niche by repulsion of cells into an environment less favourable to ISC maintenance (Figure 2).

### Peri-cryptal telocytes as gradient regulators

Telocytes were first described in 2010 by Popescu and Faussone-Pllegrini who demonstrated long (up to 1 mm) and thin (0.05–0.2 µm diameter) branched cytoplasmic processes (telopods) and a unique organelle composition by morphological analysis using transmission electron microscopy [12]. Given the relatively long distance covered by telopods and the sub-epithelial network formed between telocytes, trophoblasts and SEMFs, it is conceivable that these cells could provide a very fast and efficient communication method from the villus tip to the ISCs and ISC niche at the crypt base in order to tightly regulate cell number and activity along the entire crypt-villus axis. Telocytes can be divided into several different sub-populations and have non-uniform abundance along the crypt-villus axis. A high density of telocytes that are PDGFRα^lo^ and CD81^-^ are not thought to contribute directly to ISC maintenance but populate the crypt villus junction and the villus tip and may control BMP signalling gradients to regulate differentiation and anoikis [11]. Recently, telocytes that populate the small intestinal villus tip lamina propria were shown by single cell RNAseq to express Lgr5 (Figure 2) whereas telocytes that were found in the villus centre, villus bottom or crypt did not [89]. Several frizzled receptors (fzd1, 2, 4, and 7) have also been documented in intestinal telocyte populations [43,90]. Fzd1 has been shown to be reduced in expression in villus tip telocytes compared with crypt base telocytes demonstrating spatial expression of this family of receptors [89]. However, the functional significance of these differential expressions is currently unknown. It is intriguing to speculate that villus tip telocytes may be implicated in sensing R-spondin and Wnt ligand concentrations via Lgr5 and fzd receptors at the most distant point along the crypt-villus axis from the ISC niche and feedback accordingly along the crypt-villus axis to regulate production of Wnt signalling components. Further study is also warranted to determine whether Lgr5^+^ telocytes represent another population of mesenchymal stem cells that may help to maintain specialised mesenchymal cell numbers which will subsequently regulate crypt-villus axis gradients. It is also currently unclear as to whether a single telocyte can release different quantities of gradient regulators along its telopods or whether modulation of telocyte numbers generates the observed gradients.

### Other intestinal stem cell niche components and considerations

In addition to epithelial cells, telocytes, trophocytes and SEMFs discussed above, other cell populations have the capacity under certain conditions to contribute to ISC niche maintenance. Immune cell populations modulate the inflammatory environment in the intestine and a constant complex surveillance programme involving immune activation and tolerance helps maintain homeostasis. Macrophages have been shown to have direct contact with telocytes within the muscularis and can contribute to signalling and homeostasis [91]. Pro-inflammatory cytokine production (such as TNF) from various immune cells affects epithelial cell survival and can contribute to accelerated cell loss at the villus tip [92,93] which may feedback to accelerate cell production at the crypt base.

There are regional differences in extracellular matrix (ECM) composition along the crypt-villus axis and several components support intestinal epithelial cell turnover. In particular, collagen VI, laminins α1 and α2 are polarised around the crypt base and laminin α5 is found along the villus [94,95]. Lamin α5 was shown to be critical in maintaining villus architecture, goblet cell maturation and preventing progenitor cell hyperplasia [94]. Cell surface receptors for laminins such as the integrins also show different distributions along the crypt-villus axis. Transgenic mouse studies have demonstrated that deletion of the integrin-β4 cytoplasmic domain have reduced small intestinal proliferation and die at birth [96]. Whereas, increased small intestinal proliferation was mediated by altered Hedgehog signalling when integrin β1 was conditionally knocked out in the small intestine [97]. The success of intestinal organoid cultures is not only owed to the identification of Lgr5 ligands, co-factors and growth factors, but also to the laminin and fibronectin-rich basement membrane matrix used to support their growth. An active area of current research is in the development of a more *in vivo*-like well-defined matrix that sustains long term organoid culture and that better represents *in vivo* crypt-villus axis biology. The development of different matrices has clearly demonstrated that ISC cell morphology and function change along with Hippo signalling perturbations when cultured with matrices of different stiffnesses with softer matrices favouring differentiation and stiffer matrices supporting more proliferation [98]. The stiffness of the ECM varies upon composition, and it is therefore likely that the stiffness varies along the crypt-villus axis (Figure 2). Taken together, the ECM constituents of the basement membrane that are deposited by neighbouring mesenchymal and epithelial cells are key intestinal stem cell niche regulators and need to be considered in addition to the cellular compartments.

A very diverse population of microbiota exist within the intestinal lumen. An in-depth discussion of how different commensal and pathogenic bacteria and their metabolites may affect the stem cell niche is beyond the scope of this review, but many likely mediate their effects indirectly via modulating pathways discussed above. However, intestinal microbiota should not be ignored as ISC and crypt-villus/crypt regulators during homeostasis and disease, some activity of which may be via novel as yet unknown mechanisms [99]. The O_2_ gradient along the crypt-villus/crypt axis also regulates anaerobic microbiome composition and cellular activity [100]. O_2_ availability may therefore also help to regulate the ISC niche. Similarly, dietary nutrients modulate ISC niche activity, crypt-villus axis biology and the microbiome. For instance, L-arginine stimulates Paneth cell production of Wnt3a [101], mTOR is involved with sensing of nutritional state [102,103], calorie restriction increases Lgr5^+^ ISCs and Paneth cells and protects against radiation-induced damage [102,104], high-fat and ketogenic diets also increases Lgr5^+^ ISC cell numbers via Wnt and Notch pathway stimulation [105,106].

## Summary

Maintenance of the ISC niche is complex and consists of multiple cell types that generate signalling components, cascades and gradients along the crypt-villus axis. The foundations of our current understanding of crypt-villus axis biology were laid in the 1940s and subsequent great advances were made in mapping epithelial dynamics during homeostasis and intestinal injury by Chris Potten and associates. However, the isolation and long-term growth of intestinal organoids following the identification of Lgr5 and its ligands by Nick Barker and Hans Clevers in 2007 and the very recent technological advances of single cell RNA sequencing and high-resolution 3D imaging are rapidly advancing our understanding of the heterogeneity and coordination that is necessary from several cellular compartments to regulate ISC activity and epithelial homeostasis.

The recently demonstrated capacity of mature epithelial cells to de-differentiate to ISCs under some conditions demonstrates the importance of the ISC niche in maintaining stemness of ISCs. The niche is not only generated by cell populations contained within the niche but also by feedback mechanisms from other nearby cell populations that contribute to generating signalling gradients along the crypt-villus/crypt axes. The ISC niche is itself dynamic and the crypt-villus axis can respond quickly to changes in environment to expand or shrink the niche as appropriate to maintain a single continuous layer of epithelium that retains the ability to absorb nutrients from the lumen whilst preventing infection/invasion by abundant commensal/pathogenic microbiota. This review has highlighted the roles of several cell populations and direct regulatory mechanisms for ISC niche maintenance, however, there are other cell populations such as immune cells that reside within the epithelium and underlying lamina propria that contribute a continuous surveillance programme. Immune cell secretions can regulate the survival of intestinal epithelial cells and so by affecting cell number along the crypt-villus axis, can modulate the size and activity of the ISC niche, but discussion of these regulatory mechanisms is beyond the scope of this review.

Cellular sources and activities of Wnts and R-spondins, their antagonists and impact on the ISC niche have started to be documented but still need to be further characterised to enable a greater understanding of disease processes in the GI mucosa and the development of novel therapeutic approaches. Many studies assessing epithelial-mesenchymal interactions were performed in experimental animals owing to their dynamic nature. However, differences between ISC regulators between humans and mouse have been recognised, for instance in human colon additional extra-mucosal fibroblast-like cells have been identified as sources of Wnt2B/Rspo3 [107]. Further confirmatory studies, particularly appertaining to the newly identified telocyte/trophocyte populations are now required in the human. Regional differences in anatomy, cell populations and protein expression are found along cephalocaudal axis, for example villi become shorter and crypts get longer from proximal to distal small intestine and whilst changes in differentiated epithelial cell proportions along this axis have been well documented, differences in mesenchymal cell populations have not. Recently demonstrated heterogeneity within the telocyte populations may make this characterisation difficult.

The extracellular basement membrane matrix produced by ISC niche-associated cells is important in ISC niche maintenance *in vivo* but is also a key component of intestinal enteroid growth success. The development of an ECM capable of supporting intestinal enteroid growth that is better aligned with the normal human ISC niche-associated basement membrane and the establishment of stiffness and signalling gradients within the culture system may allow more representative *in vitro* gut co-culture models that could contribute to personalised medicine approaches and enhance our understanding about how genetic variation may modulate ISC niche activity during homeostasis and intestinal disease.

## Perspectives

- Importance: Delineating ISC niche regulation is not only fundamental to the understanding of intestinal epithelial biology and intestinal disease pathogenesis, but it also impacts on many other scientific research fields associated with drug development, drug safety, nutrition and dietary impact on health.
- Current thinking: There is a high degree of plasticity and heterogeneity within intestinal epithelial and mesenchymal compartments that has recently adjusted our knowledge of intestinal crypt-villus axis biology. The process of de-differentiation of mature epithelial cell populations to regain stem cell function and the identification of trophoblast and telocyte populations, their geographic locations and how they regulate signalling gradients to modulate the ISC niche, demonstrate clear communication mechanisms from the villus tip/crypt table to regulate the ISC niche in the crypt base.
- Future directions: Single-cell sequencing has highlighted a high degree of heterogeneity within intestinal mesenchymal cell populations, the contribution of these populations to ISC niche and crypt-villus axis homeostasis still needs resolution which will also enable more clarity in the currently evolving telocyte/SEMF nomenclature. Future studies need to address the integration of multiple signals from well-defined mesenchymal sub-populations and their communication mechanisms with different crypt-villus axis regions and cell types of the intestinal epithelium, which will help to further elucidate processes responsible for re-forming the ISC niche upon extreme mucosal damage and identify novel approaches to treat intestinal disease. Regional differences in ISC niche regulation within the small intestine, colon and between different mammalian species should also be defined.

## Open Access Statement

Open access for this article was enabled by the participation of University of Liverpool in an all-inclusive Read & Publish pilot with Portland Press and the Biochemical Society under a transformative agreement with JISC.

## Abbreviations

ECM: extracellular matrix
EGF: epidermal growth factor
EGFR: epidermal growth factor receptor
ISCs: intestinal stem cells
RSPO: R-spondin
SEMFs: sub-epithelial myofibroblasts
